# Rapid Limit Test of Seven Pesticide Residues in Tea Based on the Combination of TLC and Raman Imaging Microscopy

**DOI:** 10.3390/molecules27165151

**Published:** 2022-08-12

**Authors:** Xin Liang, Li Li, Cuiyan Han, Yan Dong, Feng Xu, Zhen Lv, Ying Zhang, Zhijie Qu, Wei Dong, Yongqiang Sun

**Affiliations:** Pharmacy School, Qiqihar Medical University, Qiqihar 161006, China

**Keywords:** Raman imaging microscope, TLC, pesticide residues, tea, limit test

## Abstract

Numerous studies have shown that pesticide residues in tea exceeding the maximum residue limits (MRL) can cause harmful effects on the human body. There are many limitations in the existing analytical methods for pesticide residues in tea, so new analytical methods need to be developed. We developed a limit test method that combines thin-layer chromatography with Raman imaging microscopy (TLC-RIM). Seven residual pesticide components in tea (Avermectin, Methomyl, Carbendazim, Imidacloprid, Chlorothalonil, Azoxystrobin, and Acetamiprid) could be preliminarily separated by TLC and then irradiated by a 532 nm laser. Raman spectra of seven pesticides obtained by Raman imaging microscopy could be used to test whether the pesticide residues in tea exceed the MRL. The limits of detection of the seven pesticides were 0.04, 0.10, 0.24, 0.20, 0.12, 0.12, and 1.0 mg/mL, respectively. The simulated positive test showed that the matrix in tea did not interfere with the test of the seven pesticides. When the pesticides were tested within 8 h, the RSD of the peak heights of the seven pesticides were 1.2%~9.6%; the test results of three batches of tea showed that the imidacloprid in one batch of tea exceeded its MRL, and the results were consistent with that by UPLC-MS/MS. The TLC-RIM is fast, sensitive, stable, specific, and reliable.

## 1. Introduction

Tea is a kind of popular beverage and is consumed by almost two-thirds of the world’s population [1]. There are many functional nutrient constituents in tea, such as theanine, vitamins, alkaloids, catechins, polysaccharides, and essential minerals [2,3,4]. In order to control pests, insecticides are frequently used, which could cause potential risks to public health [5,6]. This issue has aroused wide concern among the public. Commonly used chemical pesticides include Abamectin (A), Methomyl (B), Carbendazim (C), Imidacloprid (D), Chlorothalonil (E), Azoxystrobin (F), and Acetamiprid (G). According to the Chinese food standards, the maximum residue limits (MRL) of these pesticide residues are 0.1, 0.2, 5.0, 0.5, 10.0, 0.5, and 10.0 mg/kg, respectively [7].

In recent years, the phenomenon of excessive pesticide residues in tea is not uncommon. A typical report from European Food Safety Authority (EFSA) [8], which analyzed pesticide residue levels in food on the European market, indicated that 4.5% of overall 91,015 samples exceeded the MRL and probably posed a threat to humans. Among them, the MRL exceeding the rate for tea products had increased significantly [9,10,11]. A lot of research demonstrated that pesticides, because of their toxic properties, were associated with various health problems, such as lipid metabolism and endocrine disorders, cardiovascular disease, and negative effects on the nervous system and male reproductive system [12]. Therefore, it is essential to seek simple and fast analytical techniques for the quality control of tea.

Until now, numerous methods have been developed for pesticide determination over the past decades [13,14,15]. Conventional analytical methods for determining pesticide residues in food always rely on gas/liquid chromatography coupled with a series of selective and sensitive detectors, including high-performance liquid chromatography (HPLC), gas chromatography-mass spectroscopy (GC–MS) [16], liquid chromatography-mass spectroscopy (LC–MS) [17,18], and surface-enhanced Raman scattering (SERS) [19,20,21,22]. Despite their salient advantages of high sensitivity and accuracy in quantitative and qualitative analysis, these techniques still require complicated sample pretreatment, as well as hours or even days for the completion of the whole analysis process.

Traditional thin-layer chromatography (TLC) is a technique for the simple and rapid separation of chemical components [23]. With the help of auxiliary means such as chemical color development and ultraviolet light irradiation, the method can reflect the characteristics of a certain group in the chemical structure of the component, but the specificity is low. Raman spectroscopy studies the inelastic scattering phenomenon of compound molecules after being irradiated by light. The fingerprint structure information of compounds can be reflected by Raman spectroscopy. It is a highly specific spectral analytical technology [24,25,26,27]. The purpose of Raman imaging is to visualize the distribution of different components in a sample. Thus, each pixel in the image corresponds to a Raman spectrum that may be compared to an established Raman database or spectrum of a reference substance to determine a specific analyte or spectral background measurements in this location [28,29]. Area scanning is one approach of Raman imaging that can achieve an entire sample area being illuminated with laser light, and its spatial information is obtained in one scan without relative movement between the laser and the sample [30]. In area scanning, the single spectrum of these components can be obtained by different colors in the imaging [31,32].

The main purpose of this study was to develop a limit test method named thin-layer chromatography combined with Raman imaging microscopy (TLC-RIM), which can rapidly separate and accurately detect small amounts of pesticide residues in tea. This method can provide a reference for the rapid limit test of pesticide residues in tea.

## 2. Materials and Methods

### 2.1. Materials

All reagents were analytical grade and were bought from Merck Drugs and Co, Germany. The reference substances of A (99.2%), B (99.9%), C (99.5%), D (99.8%), E (99.6%), F (99.2%), and G (99.7%) were purchased from Dr. Ehrenstorfer GmbH (Germany). The reference substances were dissolved in methanol (99.5%). The three real samples of tea were supplied by three different manufacturers (China), and the pesticide residues were extracted by acetonitrile (99.5%). The pesticide residues were eluted by cyclohexane (99.7%), glacial acetic acid (99.0%), ethyl acetate (99.5%), anhydrous ethanol (99.7%), and triethylamine (99.0%).

TLC could be obtained by a thin-layer plate (Merck KGaA, Darmstadt, Germany) that is composed of high-performance silica gel and fluorescing additive F_254_, which is called GF_254_ thin-layer plate for short. The particle size is 8 ± 2 μm, the layer thickness is 0.2 ± 0.03 mm, and the carrier is aluminum. The microinjector (10 µL) used for spotting on thin-layer plates was purchased from Zhenhai Glass Instrument Factory, Ningbo, China.

### 2.2. Apparatus and Conditions

Separated compounds on TLC were located under 254 nm by an Ultraviolet analyzer (YOKO-2F; Wuhan YOKO Technology Ltd., Wuhan, China). Raman spectra and their imaging were obtained by use of a DXR™ xi Raman Imaging Microscope (Thermo Fisher Scientific, Waltham, MA, USA) with an excitation wavelength of 532 nm, a resolution of 5.0 cm^−1^, and a 10× long working distance microscope objective. The excitation power was 10 mW, the integration time was 0.5 s, and the number of scans was 20. The scan range was 3300–100 cm^−1^, with a 50 µm confocal pinhole DXR532 full range grating (400 line/mm). The detector was a TE-cooled electron-multiplying CCD (EMCCD). Area scanning was chosen as the scanning mode, the scanning area was more than 150 μm × 150 μm, and the total scanning time was 20 min.

Ultra-high-performance liquid chromatography-tandem mass spectrometry (UPLC-MS/MS) was operated on a Dionex UltiMate 3000 ultra-performance liquid chromatography—TSQ quantum mass spectrometer system (Thermo Fisher Scientific, USA). The limit test results of the pesticide residues in real samples were verified by the UPLC-MS/MS, and B, C, D, and E were determined by gradient elution using a Kromasil C_18_ column (100 × 2.1 mm × 1.8 μm) with a mobile phase of acetonitrile-water (containing 10 mol/L ammonium acetate); and A, F, and G were determined by gradient elution using the same column with a mobile phase of acetonitrile-water (containing 0.1% formic acid). at a flow rate of 0.3 mL/min. the column temperature was 40 °C. ESI positive-ion in MRM mode was used to monitor the precursor ion→product ion transitions *m*/*z* 890.6→305.3 (A), 163.0→87.9 (B), 191.7→159.7 (C), 255.9→174.8 (D), 244.9→181.9 (E), 404→372 (F), and 223→126 (G).

### 2.3. Solutions Preparation

According to the MRL of seven pesticide residues in tea, reference substance solutions were prepared by dissolving each pesticide (A, B, C, D, E, F, and G) in methanol to obtain a solution of 0.05, 0.10, 2.5, 0.25, 5.00, 0.25, and 5.00 mg/mL, respectively.

In order to test the separation of the seven pesticides on the TLC, the above seven reference substance solutions were used to prepare mixture reference solution M_1_ and mixture reference solution M_2_.

M_1_ was prepared by taking the appropriate amount of each of the four pesticide (A, B, C, and D) solutions together, drying with nitrogen at room temperature, then precisely adding 1 mL of methanol to the redissolution to obtain mixture solutions of 0.04 mg/mL (A), 0.10 mg/mL (B), 0.24 mg/mL (C), and 0.24 mg/mL (D), respectively.

M_2_ was prepared by taking the appropriate amount of each of the three pesticides (E, F, and G) together, drying with nitrogen at room temperature, then precisely adding 1 mL of methanol to redissolve to obtain a mixture solution of 0.24 mg/mL (E), 0.24 mg/mL (F), and 1.4 mg/mL (G), respectively.

The sample solutions were prepared as follows: weigh 50 g of tea sample, pass the finely ground powder through a 40-mesh sieve, add 12 g NaCl powder, mix well, add 200 mL of acetonitrile to dissolve, and then sonicate for 15 min. After centrifugation under 4000 rpm/min, the filtrate was obtained by passing the supernatant through a NH_2_/Carb solid phase extraction cartridge and concentrating the filtrate to about 2 mL with a rotary evaporator (75 °C), transferring it to a tiny chromatographic vial, blow drying with nitrogen, precisely adding 100 μL methanol to reconstitute it, and covering it.

The negative sample was a tea sample tested by the Qiqihar Institute for Food and Drug Control and was confirmed to be free of the seven pesticide residues (A–G). The preparation method of the negative sample solution was the same as that of the sample solution.

According to the MRL of seven pesticide residues in tea, simulated positive samples were prepared by adding the seven pesticides (A–G) reference into negative samples at the content of 0.10, 0.20, 5.00, 0.50, 10.00, 0.50, and 10.00 mg/kg, respectively.

### 2.4. The TLC

Thin-layer chromatography (TLC) is a simple and fast separation technique. In order to obtain a better separation effect, seven pesticides were separated by the following two kinds of TLC:

TLC_1_ was used to separate non-nitrile compounds: 10 μL of reference substance (A, B, C, and D) solutions and the mixture solution (M_1_) were spotted on a GF_254_ thin-layer plate (8 cm × 10 cm) at a distance of 1 cm from the bottom. The spots were eluted to a distance of 8 cm in a development chamber saturated with developing agent I [cyclohexane: glacial acetic acid: ethyl acetate: anhydrous ethanol (6:1:2:1, *v*/*v*/*v*/*v*)].

TLC_2_ was used to separate nitrile compounds: 10 μL of reference substance (E, F, and G) solutions and the mixture solution (M_2_) were spotted on a GF_254_ thin-layer plate (8 cm × 10 cm) at a distance of 1 cm from the bottom. The spots were eluted to a distance of 8 cm in a development chamber saturated with developing agent II [cyclohexane: triethylamine: ethyl acetate: anhydrous ethanol (6:1:2:1, *v*/*v*/*v*/*v*)].

Then, all the plates were removed and the developing agent on the plate was naturally evaporated. Under UV irradiation at 254 nm, the main spots on the TLC could be observed.

### 2.5. The TLC-RIM

In the study, we focused on developing a rapid and specific limit test method by TLC coupled with Raman imaging microscopy (TLC-RIM) to control seven pesticide residues in tea.

After the preliminary separation of seven pesticides by the TLC, the main spots on the TLC were observed and marked under 254 nm ultraviolet light. Then, the pesticides in the spots were enriched with methanol in situ on the GF_254_ thin-layer plate for Raman spectroscopic analysis. When the methanol on the plate was naturally evaporated, Raman images of enriched pesticides in situ could be obtained by area scanning with a 532 nm laser source under a microscope, and the Raman spectrum of each pesticide could be obtained from the corresponding Raman images. The Raman spectrum of the pesticide residues in tea could be obtained by the same method. When the pesticide residue content in tea does not exceed the MRL, the Raman spectrum should be consistent with the Raman spectrum of the corresponding reference substance solutions, but the characteristic peak heights should be lower than that of the reference substance.

## 3. Results and Discussion

### 3.1. Separation by the TLC

**Figure 1:**
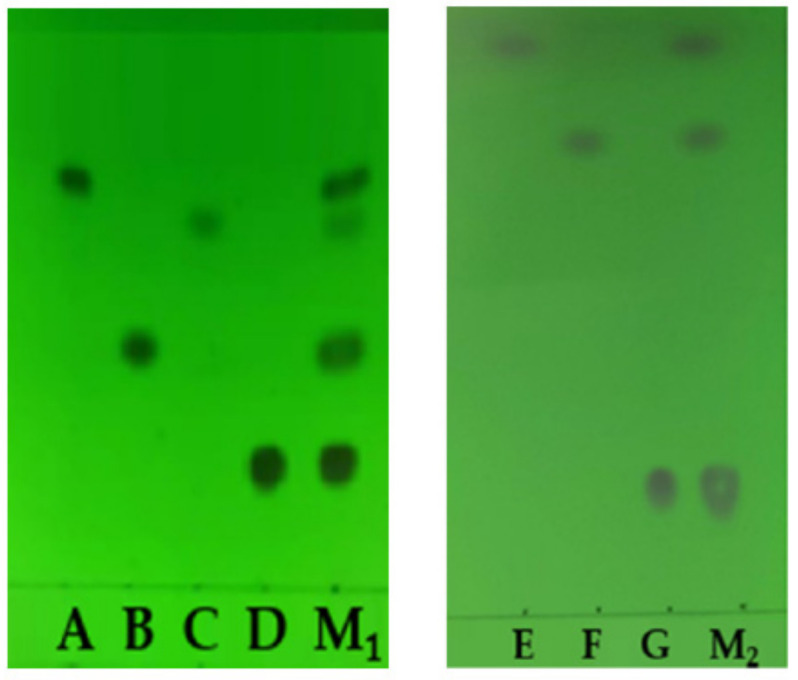
TLC of the seven pesticides and their mixture solution. (A, B, C, D, E, F, and G: Abamectin, Methomyl, Carbendazim, Imidacloprid, Chlorothalonil, Azoxystrobin, and Acetamiprid, respectively. M_1_: Mixture solution of A, B, C, and D. M_2_: Mixture solution of E, F, and G.).

The reference substance (C) solution was diluted to a concentration of 0.24 mg/mL. According to the TLC_1_, A, B, C, and D were effectively separated, and their R_f_ values were 0.76, 0.47, 0.71, and 0.24, respectively. The reference substance (E and G) solutions were diluted to the concentration of 0.24 mg/mL (E) and 1.4 mg/mL (G), respectively. According to the TLC_2_, E, F, and G were effectively separated, and the R_f_ values were 0.80, 0.66, and 0.20, respectively; the results were shown in Figure 1.

### 3.2. Limit Test by the TLC-RIM

**Figure 2:**
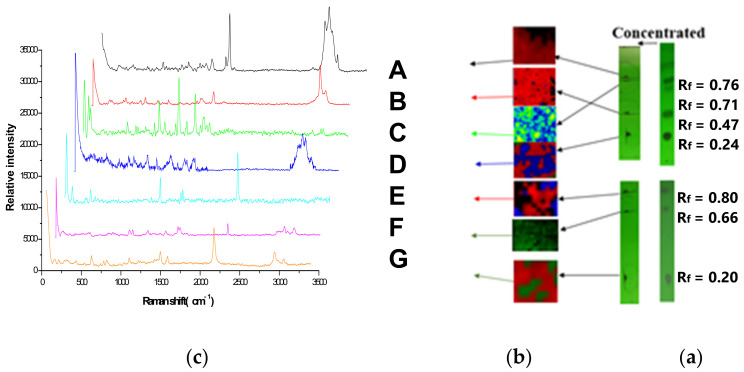
The Raman spectra of the seven pesticides in mixture by TLC-RIM. ((**a**–**c**): The TLC, Raman imaging, and Raman spectra of the seven pesticides. A, B, C, D, E, F, and G: Abamectin, Methomyl, Carbendazim, Imidacloprid, Chlorothalonil, Azoxystrobin, and Acetamiprid, respectively).

According to the TLC-RIM, the pesticides in the mixture solution on the TLC (Figure 1) were marked and enriched under a 254 nm ultraviolet light, then Raman imaging and corresponding spectra were acquired in Figure 2. The Raman spectrum of A (R_f_ = 0.76) was acquired by the black region, B (R_f_ = 0.47) by red, C (R_f_ = 0.71) by green, D (R_f_ = 0.24) by blue, E (R_f_ = 0.80) by red, F (R_f_ = 0.66) by dark green, and G (R_f_ = 0.20) by red.

### 3.3. The Influence of GF_254_ and the Developing Agents on the Raman Spectra of the Pesticides

Raman spectrometry is a spectral analysis technology with good specificity. If the method is used as a detection method for seven pesticides separated by TLC, it should be proved that the stationary phase (GF_254_) of the thin-layer plate and developing agents (M_1_ and M_2_) will not interfere with the main characteristic peaks of the Raman spectrum of the seven pesticides. Therefore, the following experiments and comparisons were carried out in this paper.

**Figure 3:**
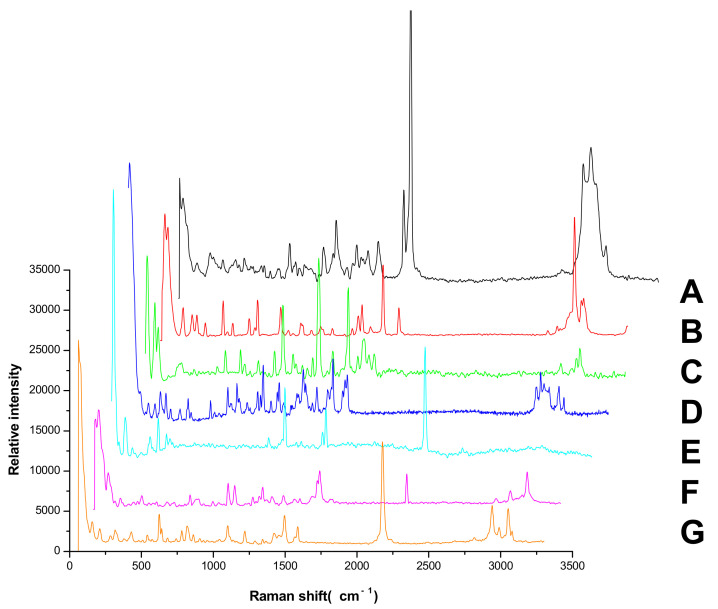
The Raman spectra of seven pesticide reference powders. (A, B, C, D, E, F, and G: Abamectin, Methomyl, Carbendazim, Imidacloprid, Chlorothalonil, Azoxystrobin, and Acetamiprid, respectively).

The Raman spectra of the seven pesticide reference powders were detected directly; the results were shown in Figure 3. The Raman spectra by TLC-RIM were shown in Figure 2c. The Raman shift value and relative peak intensities of the Raman spectrum characteristic peaks of the seven pesticides were listed in Table 1.

When the Raman spectra of reference substances detected by TLC-RIM were compared with the spectra of the corresponding reference powders, the results showed that for each pesticide, the Raman shift values (cm^−1^) of the characteristic peaks obtained by TLC-RIM were basically the same as the Raman shift values (cm^−1^) of the characteristic peaks obtained from the Raman spectrum of the powders, but the relative peak intensities of most pesticides by TLC-RIM decreased slightly. For pesticides A, B, C, and D, we selected the peaks (ν_CH_) as the reference peak and calculated the relative heights of the other peaks. For pesticides E, F, and G, we selected the peaks (ν_-C≡N_) as the reference peak and calculated the relative heights of other peaks. In the Raman spectrum of A by TLC-RIM, the relative peak intensities of ν_C=C_ (1674~1626 cm^−1^), β_CH3_ (1450 cm^−1^), ν_C-C_ (1157 cm^−1^), and γ_=CH_ (838 cm^−1^) became weaker. In the Raman spectra of B, D, E, and G, almost all the characteristic peak intensities became weaker; in the Raman spectra of C and F, the intensities of characteristic peaks were almost unchanged except the peak (3069 cm^−1^) from ν_=CH_ became weaker.

It could be seen that the Raman spectra obtained by TLC-RIM and the spectra obtained from the powder for the seven pesticides had an obvious correlation, so the TLC-RIM could be used for separation and the limit test of the seven pesticide residues in tea.

### 3.4. Analysis of Characteristic Peaks of Seven Pesticides

According to whether the chemical structures of the seven pesticides contain the -C≡N group, they can be divided into the following two categories:

Non-nitrile compounds: there are no -C≡N groups in the structures of A, B, C, and D. It can be seen from Table 1 that there are no benzene rings and double bonds in the chemical structure of B, so there is no signal peak (ν_=CH_) at 3100–3000 cm^−1^ in its Raman spectrum. This feature can be used to distinguish B from A, C, and D. There are five -C=C- bonds in the structure of A, while there are only three -C=C- bonds in C and two -C=C- bonds in D, so in the spectrum of A, the peak of ν_C=C_ at 1674 cm^−1^ is the strongest; while the peak of ν_C=C_ is a quartet peak at 1544~1654 cm^−1^ in the spectrum of C, and the peak of ν_C=C_ is a double peak at ~1586 cm^−1^ in the spectrum of D, So A can be distinguished from C and D by the feature. The chemical structure of C contains five C-N bonds, three C-C bonds, one methyl group, and one disubstituted benzene ring, so the ν_C-N_ (1267 cm^−1^) peak is the strongest, which can be used to distinguish C from D. At the same time, the chemical structure of D contains three ν_CH2_ and three ν_=CH_ within phenyl rings in different chemical environments, so C and D can also be distinguished by six-tuplets at 3094~2944 cm^−1^.

Nitrile compounds: there are -C≡N groups in the structures of E, F, and G. It can be seen from Table 1 that E and F are aromatic nitriles, and the characteristic peaks from ν_-C≡N_ are at 2242 cm^−1^ and 2230 cm^−1^; G is unsaturated nitrile, and the characteristic peak from ν_-C≡N_ is at 2177 cm^−1^. Since there are no methyl group, methylene group, or aromatic hydrogen in the chemical structure of E, there are no signal peaks at 3100–3000 cm^−1^ and 3000–2850 cm^−1^, which can be used to distinguish E from F and G. In the spectrum of G, there is a characteristic peak from ν_C-Cl_ (624 cm^−1^), which can be used to distinguish F from G. At the same time, the peak intensity ratio of ν_-C≡N_ (2230 cm^−1^) and ν_C=C_ within phenyl rings (~1624 cm^−1^) is 10:8 in the spectrum of F, while the peak intensity ratio of ν_-C≡N_ (2177 cm^−1^) and ν_C=C_ within phenyl rings (1590~1496 cm^−1^) is 10:2.5 in the spectrum of G, so this difference can also be used to distinguish F from G.

In conclusion, the characteristic peaks of the Raman spectra of the seven pesticides are significantly different, and the TLC-RIM method has high specificity.

### 3.5. Experiment of Simulated Positive Samples

**Figure 4:**
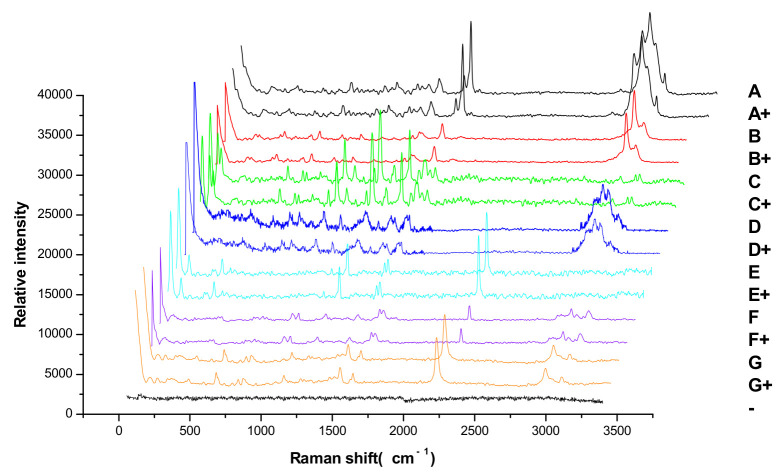
Raman spectra of the seven pesticides in the simulated positive sample by TLC-RIM (A, B, C, D, E, F, and G: Abamectin, Methomyl, Carbendazim, Imidacloprid, Chlorothalonil, Azoxystrobin, and Acetamiprid, respectively. A^+^, B^+^, C^+^, D^+^, E^+^, F^+^, G^+^: Simulated positive samples containing A, B, C, D, E, F, and G;−: Negative sample).

In total, 10 μL of reference substance solutions, simulated positive sample solutions, and negative sample solution were deposited onto the GF_254_ thin-layer plate, respectively. The experiment was carried out by the TLC-RIM, and the result was shown in Figure 4. The major spots of the simulated positive sample were in the same position as the corresponding reference substance when no spots were observed in the negative sample on the TLC. The result indicated that matrix compositions in tea did not interfere with the observation of the pesticides on the TLC. At the same time, the Raman spectra of the simulated positive samples were also in accordance with the corresponding reference substances when no Raman signal was obtained in the negative sample. The result further confirmed that the matrix in tea did not interfere with the limit test of the seven pesticide residues. It showed that the TLC-RIM has strong specificity.

### 3.6. Stability Test

After simulated positive sample solutions were placed for 0, 1, 2, 4, and 8 h, the Raman spectra of A, B, C, D, E, F, and G in the solutions at different times were detected by the TLC-RIM. The relative standard deviation (RSD) values of the peak heights from each pesticide (1.2~8.1%, 1.6~9.2%, 1.2~5.4%, 1.5~9.6%, 1.4~7.6%, 2.3~8.3%, and 1.2~7.5%, respectively) indicate that the method has good stability.

### 3.7. Inspection of the Limit of Detection

**Figure 5:**
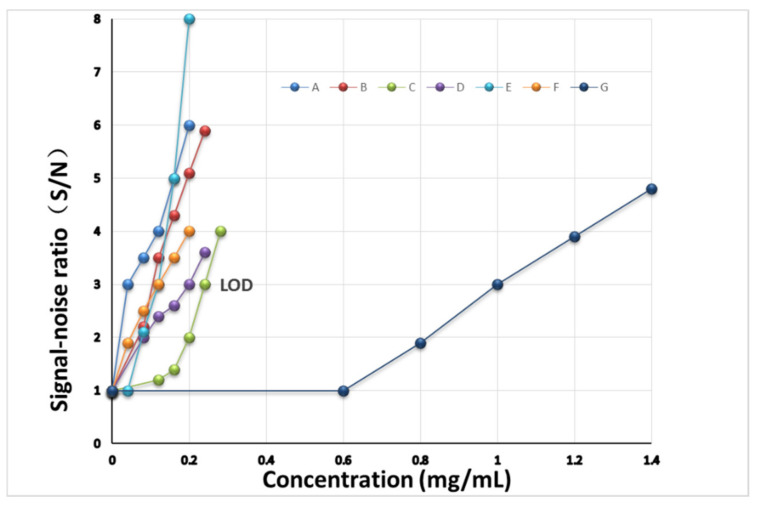
LOD of the seven pesticides by TLC-RIM. (A, B, C, D, E, F, and G: Abamectin, Methomyl, Carbendazim, Imidacloprid, Chlorothalonil, Azoxystrobin, and Acetamiprid, respectively).

According to the preparation method of the sample solution and the MRL of the seven pesticide residues in tea, the reference substance solutions were diluted with methanol at a concentration of 0.04~0.20 mg/mL, which is equivalent to 0.08~0.40 mg of pesticide (A, E, and F) per kilogram of tea; 0.08~0.24 mg/mL, which is equivalent to 0.16~0.48 mg of pesticide (B and D) per kilogram of tea; 0.12~0.28 mg/mL, which is equivalent to 0.24~0.56 mg of pesticide (C) per kilogram of tea; and 0.60~1.40 mg/mL, which is equivalent to 1.20~2.80 mg of pesticide (G) per kilogram of tea. A total of 10 μL of the solutions with different concentrations were deposited onto GF_254_ thin-layer plates, respectively, and the corresponding Raman spectra were obtained by the TLC-RIM. The results were shown in Figures S1–S7. The limit of detection (LOD) of the pesticide was the concentration for which the signal-to-noise ratio was equal to 3:1 (S/N = 3). The S/N was calculated by the characteristic peak heights at 838 cm^−1^ (A), 726 cm^−1^ (B), 621 cm^−1^ (C), 636 cm^−1^ (D), 391 cm^−1^ (E), 724 cm^−1^ (F), and 628 cm^−1^ (G). The calibration curves were established by the concentration and the *S*/*N*, the results were shown in Figure 5, and the LOD of each pesticide was observed by the curve. The LODs of A, B, C, D, E, F, and G were 0.04 mg/mL, 0.10 mg/mL, 0.24 mg/mL, 0.20 mg/mL, 0.12 mg/mL, 0.12 mg/mL, and 1.00 mg/mL, respectively. In addition, when the concentration of each pesticide is greater than its LOD, the height of its Raman spectrum characteristic peaks also increases with the increase in the concentration. So, we can determine whether the amount of pesticide residue exceeds the MRL by comparison with the characteristic peak heights of the pesticide residue in tea with that of the corresponding reference solution.

According to the preparation method of the sample solution, the pesticide residue is extracted from 50 g (0.050 kg) of tea and then soluted with 100 µL (0.1 mL) of methanol. The calculation formula of the MRL:

MRL (mg/mL) = MRL (mg/kg) × 0.05 kg/0.1 mL = MRL (mg/kg)/2

Similarly, if the concentration (mg/mL) is replaced by the content (mg/kg), the calculation formula of the LOD:

LOD (mg/kg) = LOD (mg/mL) × 0.1 mL/0.050 kg = LOD (mg/mL) × 2

The comparison between the MRL and the LOD of the seven pesticide residues in tea was shown in Table 2. The results showed that the LOD is less than or equal to the MRL. Therefore, the TLC-RIM can be used as a limit test method for the pesticides that may residue in tea.

### 3.8. Limit Test of Real Samples

**Figure 6:**
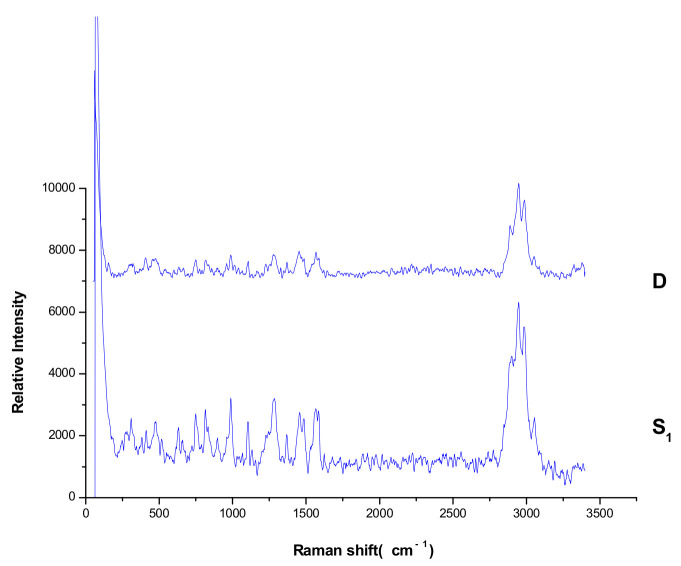
Raman spectrum of sample 1 by TLC-RIM. (S_1_: sample 1, D: Imidacloprid).

Three batches of different varieties of tea were taken, and three sample solutions were prepared. According to the TLC-RIM, 10 μL of reference substance (A, B, C, D, E, F, and G) solutions and three sample solutions were separated by TLC_1_ and TLC_2_, respectively. There were no spots from sample 2 or sample 3 observed on the TLC and there was only one spot in sample 1 at the same position as the reference substance D on the TLC, and the diameter of the sample spot is larger than that of the reference substance D the Raman spectrum of the component in the spot of the sample was almost the same as that of the reference substance D, and the characteristic peak heights from the sample were higher than that of the reference substance D. The result was shown in Figure 6. This phenomenon showed that the content of imidacloprid (D) exceeds the MRL (0.5 mg/kg) in sample 1. The above experimental results of the three tea samples were the same as those of UPLC–MS/MS, which indicated that the limit test method by TLC-RIM is accurate and reliable.

## 4. Conclusions

The study established a method (TLC-RIM) for the limit test of the seven pesticides in Tea, which has high sensitivity, good stability, and strong specificity. In addition, the method is also simple and fast.

Raman spectra of seven pesticides acquired by TLC-RIM had a good correlation with the corresponding spectra of the references. It was shown that Raman spectra of the seven pesticides were significantly different. By the simulated positive test, it was confirmed that the matrix components in tea would not interfere with the limit test of the seven pesticides. The experimental results of three real samples further proved that the specificity of TLC-RIM is stronger, and the results were verified by UPLC–MS/MS, indicating that the method is accurate and credible. In conclusion, this method can provide a new reference for the rapid limit test of the seven pesticide residues in tea.

## Figures and Tables

**Table 1:** The Assignments of Raman spectral characteristic peaks of the seven pesticides.

Chemical Structure	Raman Shift Value (cm^−1^) of Reference Powders (Relative Peak Intensity)	Raman Shift Value (cm^−1^) by TLC-RIM (Relative Peak Intensity)	Assignments
Abamectin (A) 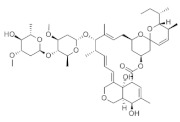	3035 (0.4)	3045 (0.4)	ν_=CH_
	2968 (1.0)	2982 (1.0)	ν^as^_CH3_
	2928 (1.3)	2946 (1.3)	ν^as^_CH2_
	2876 (1.2)	2894 (1.2)	ν^s^_CH3_
	1674 (2.9)~1626 (0.9)	1674 (1.3)~1626 (0.3) *	ν_C=C_
	1450 (0.5)	1450 (0.4)	β_CH3_
	1156 (0.7)	1157(0.3) *	ν_C-C_
	834 (0.5)	838(0.2) *	γ_=CH_
Methomyl (B) 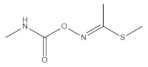	2995 (1.0)	2996 (1.0)	ν^as^_CH3_
	2932 (3.0)	2931 (2.8) *	ν^s^_CH3_
	1709 (1.0)	1709 (0.2) *	ν_C=O amide I band_
	1599 (2.0)	1593 (1.0) *	β_NH amide II band_
	~1453 double (1.0)	~1453 double (0.5) *	β_CH3_
	887 (1.0)	881 (0.4) *	ν_C-N-C_
	726 (1.2)	726 (0.5) *	ν_C-S_
Carbendazim (C) 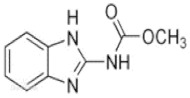	3085 (1.6)~3065 (1.2)	3087 (1.2)~3060 (1.2) *	ν_=CH within phenyl rings_
	2954 (1.0)	2954 (1.0)	ν_CH3_
	1544~1656 quartet (2.3)~	1544~1654 quartet (2.3)	ν_C=C within phenyl rings_
	1473 (5.1)	1473 (5.1)	β_CH3_
	1267 (6.9)	1267 (6.9)	ν_C-N_
	1018 (4.6)	1018 (4.6)	ν_C-C_
	728~624 (2.0)	726~621 (1.9)	γ_=CH within phenyl rings_
Imidacloprid (D) 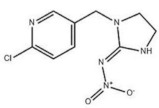	3094 (0.4), 3063 (0.7)	3094 (0.4), 3059 (0.1) *	ν^s^_=CH within pyridine rings_
	~2935 (1.0) quartet	~2944 (1.0) quartet *	ν_CH2_
	~1584 double (1.0)	~1586 double (0.4) *	ν_C=C within pyridine rings_
	1484 (1.3)	1484 (0.4) *	β_CH2_
	1277 (1.1)	1276 (0.5) *	ν_C-N_
	1113~998 (1.2)	1115~998 (0.3) *	ν_C-C_
	821~758 (0.5)	818~753 (0.3) *	γ_=CH within pyridine rings_
	~636 (0.1)	~636 (0.06) *	ν_C-CI_
Chlorothalonil (E) 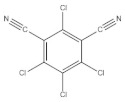	2241 (1.0)	2242 (1.0)	ν_-C≡N_
	1553~1533 double (0.4)	1551~1530 double (0.2) *	ν_C=C_
	1265~1156 double (0.6)	1265~1152 double (0.5) *	ν_C-C_
	391 (0.3)	391 (0.2) *	
Azoxystrobin (F) 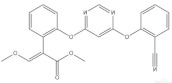	3069 (1.0)	3069 (0.3) *	ν_=CH within phenyl rings_
	2951 (0.4)	2956 (0.4)	ν^as^_CH3_
	2855 (0.2)	2854 (0.2)	ν^s^_CH3_
	2230 (1.0)	2230 (1.0)	ν_-C≡N_
	~1624 double (1.0)	~1624 double (0.8)	ν_C=C within phenyl rings_
	1227 (0.5)	1227 (0.4)	β_=CH within phenyl rings_
	1037~990 double (0.6)	1040~995 double (0.5)	ν_C-C within phenyl rings_
	727 (0.1)	724 (0.1)	γ_=CH within phenyl rings_
Acetamiprid (G) 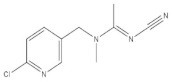	3057 (0.3)	3052 (0.2) *	ν_=CH within phenyl rings_
	2943 (0.4)	2941 (0.3) *	ν^as^_CH3_
	2177 (1.0)	2177 (1.0)	ν_-C≡N_
	1587~1502 double (0.3)	1590~1496 double (0.25) *	ν_C=C within phenyl rings_
	1427 (0.1)	1427 (0.06) *	β_CH2 within phenyl rings_
	1222 (0.1)	1224 (0.06) *	ν_C-N_
	1106 (0.2)	1106 (0.1) *	ν_C-C_
	819~784 double (0.3)	822~782 double (0.2) *	γ_=CH within phenyl rings_
	~625 double (0.3)	~628 double (0.2) *	ν_C-CI_

ν, stretching vibration; β, bending vibration. γ, out-of-plane bending vibration. *, The relative intensity of the peak decreased at the same Raman shift values as the reference powders. The unique peaks in the Raman spectra of the seven pesticides were marked in red.

**Table 2:** The comparison between MRL and LOD of the seven pesticide residues in tea.

Drug	MRL (mg/kg)	LOD (mg/kg)	MRL (mg/mL)	LOD (mg/mL)
Abamectin (A)	0.1	0.08	0.05	0.04
Methomyl (B)	0.2	0.20	0.10	0.10
Carbendazim (C)	5.0	0.48	2.50	0.24
Imidacloprid (D)	0.5	0.40	0.25	0.20
Chlorothalonil (E)	10.0	0.24	5.00	0.12
Azoxystrobin (F)	0.5	0.24	0.25	0.12
Acetamiprid (G)	10.0	2.00	5.00	1.00

## Data Availability

Not applicable.

## Supplementary Materials

The following supporting information can be downloaded at: https://www.mdpi.com/article/10.3390/molecules27165151/s1, Figures S1–S7. Figure S1: The Raman spectra of Abamectin at different concentration; Figure S2: The Raman spectra of Methomyl at different concentration; Figure S3: The Raman spectra of Carbendazim at different concentration; Figure S4: The Raman spectra of Imidacloprid at different concentration; Figure S5: The Raman spectra of Chlorothalonil at different concentration; Figure S6: The Raman spectra of Azoxystrobin at different concentration; Figure S7: The Raman spectra of Acetamiprid at different concentration.

## Sample Availability

Samples of the compounds are available from the authors.
