# Supplementary material for: Rapid Limit Test of Seven Pesticide Residues in Tea Based on the Combination of TLC and Raman Imaging Microscopy
Source: Molecules. 2022 Aug 12;27(16):5151. doi: 10.3390/molecules27165151 (PMC9413101; doi:10.3390/molecules27165151)
Supplement: Supplementary file 1 [file molecules-27-05151-s001.zip › molecules-1803858-supplementary.pdf]

Supplementary Materials

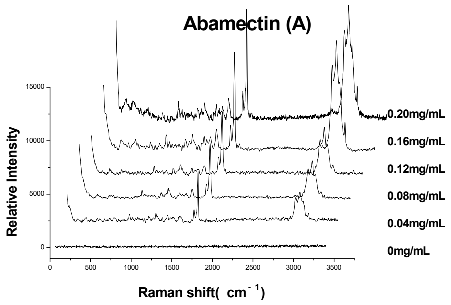


**Figure S1.** The Raman spectra of Abamectin at different concentration.

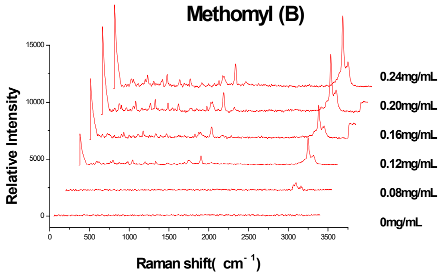


**Figure S2.** The Raman spectra of Methomyl at different concentration.

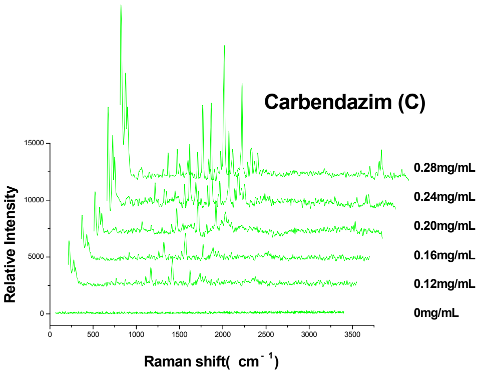


**Figure S3.** The Raman spectra of Carbendazim at different concentration.

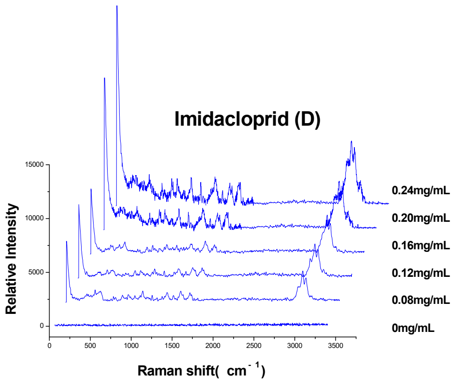


**Figure S4.** The Raman spectra of Imidacloprid at different concentration.

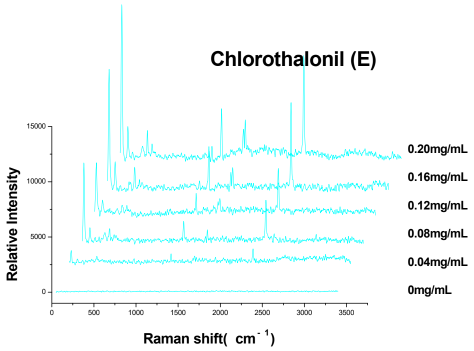


**Figure S5.** The Raman spectra of Chlorothalonil at different concentration.

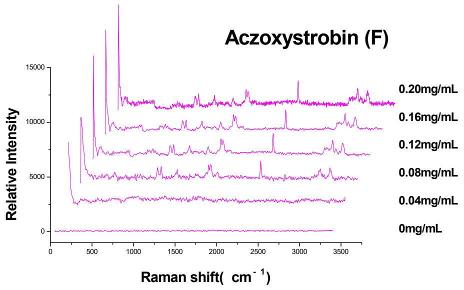


**Figure S6.** The Raman spectra of Azoxystrobin at different concentration.

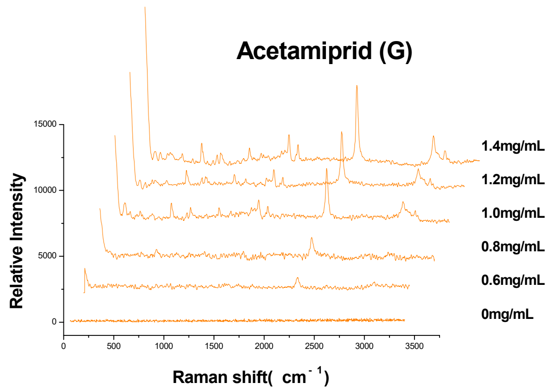


**Figure S7.** The Raman spectra of Acetamiprid at different concentration.
